# Temporal Reliability and Lateralization of the Resting-State Language Network

**DOI:** 10.1371/journal.pone.0085880

**Published:** 2014-01-24

**Authors:** Linlin Zhu, Yang Fan, Qihong Zou, Jue Wang, Jia-Hong Gao, Zhendong Niu

**Affiliations:** 1 School of Computer Science and Technology, Beijing Institute of Technology, Beijing, China; 2 Center for MRI Research and Beijing City Key Lab for Medical Physics and Engineering, school of physics, Peking University, Beijing, China; 3 Center for Cognition and Brain Disorders, Hangzhou Normal University, Hangzhou, 310015, China; 4 McGovern Institute for Brain Research, Peking University, Beijing, China; University of Helsinki, Finland

## Abstract

The neural processing loop of language is complex but highly associated with Broca's and Wernicke's areas. The left dominance of these two areas was the earliest observation of brain asymmetry. It was demonstrated that the language network and its functional asymmetry during resting state were reproducible across institutions. However, the temporal reliability of resting-state language network and its functional asymmetry are still short of knowledge. In this study, we established a seed-based resting-state functional connectivity analysis of language network with seed regions located at Broca's and Wernicke's areas, and investigated temporal reliability of language network and its functional asymmetry. The language network was found to be temporally reliable in both short- and long-term. In the aspect of functional asymmetry, the Broca's area was found to be left lateralized, while the Wernicke's area is mainly right lateralized. Functional asymmetry of these two areas revealed high short- and long-term reliability as well. In addition, the impact of global signal regression (GSR) on reliability of the resting-state language network was investigated, and our results demonstrated that GSR had negligible effect on the temporal reliability of the resting-state language network. Our study provided methodology basis for future cross-culture and clinical researches of resting-state language network and suggested priority of adopting seed-based functional connectivity for its high reliability.

## Introduction

Broca's and Wernicke's areas are two major regions associated with language processing (see review [1]). Broca's area has been traditionally linked to the language production since Pierre Paul Broca reported impairments of this region in two patients with language production dysfunction in 1861 [2]. The damage of Broca's area has been shown to result in Broca's aphasia [3]. Wernicke's area is another important cortical region in language network. Patients with Wernicke's aphasia are able to speak with normal grammar, syntax, rate, intonation, and stress, but unable to understand language in its written or spoken form [4]. Broca's and Wernicke's areas are involved in a neural language processing loop [5] and locate at different end of this loop. This neural model of language processing has been demonstrated by many studies (see review [6]), and it is also shown that more regions are correlated with this neural processing model besides Broca's and Wernicke's areas [7]–[9].

Functional connectivity (FC) of resting-state fMRI (RS-fMRI) data is rapidly emerging as a highly efficient and powerful tool for in vivo mapping of neural circuitry in the human brain. Resting-state functional connectivity (RSFC) approaches evaluate the correlation patterns of low-frequency fluctuations in the resting-state BOLD signal and generate highly detailed FC maps of complex functional systems [8], [10], [11]. A recent RS-fMRI study asserted the value of the brain spontaneous activity in language network [12]. RSFC has been used to identify the functional organization of the language [7], [9]. It is demonstrated that resting-state network is a reliable tool to access language-related networks in clinical settings [13]. It is reported that stuttering subjects showed deficits in RSFC within multiple functional systems including motor, language, auditory and default-mode networks and RSFC between these systems [14]. Moreover, many studies have identified reorganization of resting-state language network in therapy and development research [15]–[18]. A recent study evaluated the RSFC of language networks and its reliability across subjects and institutions [8]. Despite the increased appreciation and application of low-frequency oscillations in RS-fMRI data, the temporal reliability of resting-state language network is still unknown.

A fundamental feature of the human brain is the presence of both structural and functional asymmetries between two hemispheres at both macroscopic and microscopic dimensions [19], [20]. The brain asymmetry can even be observed in prenatal period, and it is always associated with successful development [20], [21]. Previous studies have indicated that asymmetries are also considered to be linked with evolution and heredity [22], [23]. Abnormal hemispheric lateralization has been shown to exist in neuropsychiatric disorders, such as autism, Alzheimer Disease and schizophrenia [24]–[26]. One of the earliest observations of brain asymmetry was the dominance of the left hemisphere in language. Broca and Wernicke both found the left lateralization of language function from lesion studies [2], [27]. Moreover, morphological asymmetries of planum temporale and pars opercularis have been described, which provides structural evidence of functional lateralization of language [24], [28], [29]. In addition, fibers between Broca's and Wernicke's areas seem to be leftward asymmetry [30]. Disrupted asymmetries in language associated cortical areas have been found not only in language related diseases, such as developmental dyslexia, but also in some psychiatric diseases, such as autism and schizophrenia [24], [31]–[34]. Functional asymmetry of language network was testified to be consistent among various research centers [8], but its temporal reliability, which should be more crucial to clinical applications, still needs to be investigated.

Additionally, in RS-fMRI studies, global signal regression (GSR), a widely used preprocessing step, has been shown to introduce negative correlations in standard fMRI analysis [35], [36] and can fundamentally alter interregional correlations within a group, or their differences between groups [37]. Chen and colleagues proposed a method to quantify global noise levels and a criterion to determine whether to include or exclude the global signal regression [38]. However, whether or not performing GSR is still controversial in the RS-fMRI field. In previous reliability related studies, many of them included GSR [11], [39], [40]. Interestingly, all those studies demonstrated high reliability of RSFC, which suggests GSR would not significantly impact the reliability pattern. However, up to now, no study directly explores the effects of GSR on reliability of RSFC.

The present work provides a temporal reliability examination of resting-state language network. Firstly, we computed RSFC maps with the seeds located at the Broca's and Wernicke's areas separately. The Intra-class correlation (ICC) was then used to evaluate the test-retest (TRT) temporal reliability of the resting-state language network. Furthermore, we investigated the functional asymmetry of resting-state language network and its TRT reliability. In addition, the impact of GSR on the temporal reliability of the resting-state language network was explored.

## Materials and Methods

### Participants

A RS-fMRI data set including 25 right-handed participants (mean age 29.44±8.47 years, 10 males) that is publicly available at NITRC (http://www.nitrc.org/projects/nyu_trt/) was used in the current study. All participants had no history of psychiatric or neurological illness, as confirmed by clinical assessment. Informed consent was obtained prior to participation. Data were collected according to protocols approved by the institutional review boards of New York University (NYU) and the NYU School of Medicine. This data set has been used to examine the TRT reliability of cross-correlations [11], ICA and Dual regression [41], amplitude of low-frequency oscillations [42], graph-theoretic network properties [39], network centrality [40], and quantifying temporal correlations [43].

### Data acquisition

A Siemens Allegra 3-T scanner was used to obtain three resting-state scans for each participant. Each scan consisted of 197 contiguous EPI functional volumes (TR  = 2000 ms; TE  = 25 ms; flip angle  = 90°, 39 slices, matrix  = 64×64; FOV  = 192 mm; acquisition voxel size  = 3×3×3 mm^3^). Scans 2 and 3 were conducted in a single scan session, 45 min apart, and were 5–16 months (mean 11±4 months) after Scan 1. During the scan, participants were instructed to rest with their eyes open.

### Image preprocessing

The preprocessing was carried out by using Data Processing Assistant for RS-fMRI (DPARSF) [44] which is based on Statistical Parametric Mapping (SPM8, http://www.fil.ion.ucl.ac.uk/spm) and RS-fMRI Data Analysis Toolkit (REST, http://www.restfmri.net) [45]. The preprocessing for each functional scan included 1) slice timing with 39^th^ slice as reference slice; 2) realignment; 3) normalization by using EPI template and resampling to 3 mm isotropic voxels; 4) spatial smoothing via a Gaussian kernel with FWHM  = 6 mm; 5) removal of linear trend and band-pass temporal filtering (0.01–0.1 Hz); and 6) regressing out nine nuisance covariates (mean signals of whole brain, white matter and cerebrospinal fluid, and six motion parameters); and 7) temporal “scrubbing” of volumes within each subject's fMRI time series that were associated with sudden head motion [46], [47]. For each subject, fMRI volumes were censored if framewise displacement (FD) of head position, calculated as the sum of the absolute values of the derivatives of the realignment estimates, was above 0.5. Additional one volume before and two volumes after that “bad” volume were censored [47]. Two subjects were excluded because the remaining data were less than four and a half minutes after temporal “scrubbing”. To evaluate the effect of the global signal regression, similar preprocessing steps were performed except that only eight nuisance covariates (mean signals of white matter and cerebrospinal fluid, and six motion parameters) were regressed out.

### Resting-state Functional connectivity and asymmetry

**Figure 1:**
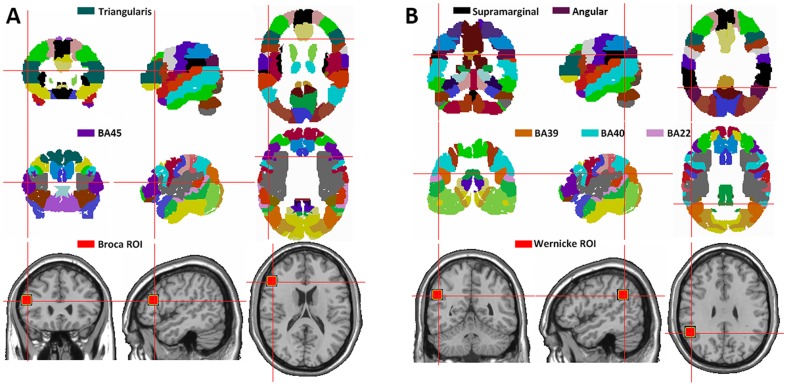
Location of seed ROIs adopted in the present study. Location of seed ROIs for Broca's (A) and Wernicke's areas (B) in comparison to the regions in AAL and BA templates.

As we focused on the cortical regions which functionally correlated with Broca's and Wernicke's areas, we estimated the RSFC maps for each subject and each functional scan using the standard seed-based correlation analysis. In line with a previous study [8], two regions of interest (ROIs) [A 5×5×5 voxels cubic ROI centered at (−51, 27, 18) mm and the other with the same size centered at (−51, −51, 30) mm] were selected as seeds (Fig. 1) to represent Broca's (lBro) and Wernicke's areas (lWer) using probabilistic atlases of human brain anatomy [29], [48]. The seed representing Broca's area was selected near the center of mass of the left pars triangularis (BA 45) and the one representing Wernicke's area was selected in the left supramarginal gyrus (at the boundaries of BA 39, 40 and 20). To estimate the functional asymmetry of language network, the left-right-flipped regions of the above two ROIs were defined as right Broca's area (rBro) and right Wernicke's area (rWer), respectively. As a result, four seed ROIs were generated. RSFC maps were produced using DPARSF [44] through computing the Pearson's correlation coefficient between each seed ROI and every voxel across the brain for each functional scan. Furthermore, the FC maps were converted to *z*-maps through Fisher's transformation (
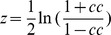
) to approach a normal distribution. After the RSFC maps were computed for each subject, group-level analysis was carried out using REST [45]. One-sample *t*-test for each functional scan was established for each seed ROI and clusters with corrected *p_FWE_* <0.05 (uncorrected *p*<0.005, voxel size > = 200) were considered significant, and masks of significant correlated regions with each ROI were generated.

The asymmetry of RSFC in the present study was defined in line with previous studies [49]. For instance, when investigating the asymmetry of the RSFC profiles of the Broca's area, the comparison of RSFC profiles between lBro and left hemisphere (LH) and those between rBro and right hemisphere (RH) was conducted to reveal the asymmetry of ipsilateral hemisphere. The comparison of RSFC of lBro-RH and that of rBro-LH was used to reveal the contralateral hemispheric asymmetry. To obtain hemispheric asymmetry of RSFC profiles, individual *z*-maps of the rBro and rWer were left-right flipped. The asymmetry index (AI) was defined as *z*-transformed RSFC of left ROI minus left-right flipped RSFC of the right ROI, which can be shown as Eq. (1).
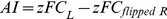
(1)


As a result, the left hemisphere of AI map revealed ipsilateral asymmetry, while contralateral asymmetry was demonstrated in right hemisphere. AI maps of both Broca's and Wernicke's areas were computed voxel by voxel across all subjects and averaged among three scans for each subject. One-sample *t*-test was conducted on individual AI maps to reveal regions which showed significant hemispheric asymmetry and were corrected for multiple comparisons *p_FWE_* <0.05 (uncorrected *p*<0.005, voxel size > = 200). Before correction, *t*-maps were masked by an “OR” mask which were generated by merging corrected RSFC maps of left and right seed ROIs.

### Test-retest reliability analysis

To assess the TRT reliability of those seed-based RSFC maps and AI maps, we calculated ICC values, a common coefficient assessing TRT reliability [50]. Take RSFC of Broca's area as an example, for each voxel, the RSFC values across the 25 subjects were reshaped into two 25×2 matrices. The two matrices can represent Scan 2 and Scan 3 (for the short-term or intra-session reliability), or Scan 1 and the mean value of Scan 2 and Scan 3 (for the long-term or inter-session reliability). Using a one-way ANOVA on each of the two matrices, we calculated the between-subject mean square (MS_b_) and within-subject mean square (MS_w_), and then ICC values were computed according the following formula:
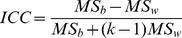
(2)k is the number of observations per subject, and k = 2 in this study. Eq. (2) implies that the higher the ICC value is, the lower the within-subject variance relative to between-subject variance will be, which means more reliable of the result. To estimate significantly reliable networks, ICC values were transformed to *Z* scores:
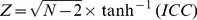
(3)where N is the number of participants. The deviation of Eq. (3) can be found in Text S1. Multiple comparisons were corrected at cluster level with pFWE <0.05 [uncorrected p<0.005 (corresponding to Z>2.81 or ICC >0.547), cluster size > = 200]. As a result, both intra- and inter-session reliability of RSFC and functional asymmetry of Broca's and Wernicke's areas were obtained.

### The evaluation of the impact of global signal regression

To evaluate the impact of global signal regression on ICC of resting-state language network, we adapted Eq. (3) to compare the ICC values obtained with GSR or without GSR [51].
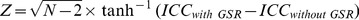
(4)where N is the number of participants as in Eq. (3). Multiple comparisons were corrected at cluster level with *p_FWE_* <0.05 [uncorrected *p*<0.005 (corresponding to *Z*>2.81), cluster size > = 200].

## Results

### Voxel-wise functional connectivity of the resting-state language network

**Figure 2:**
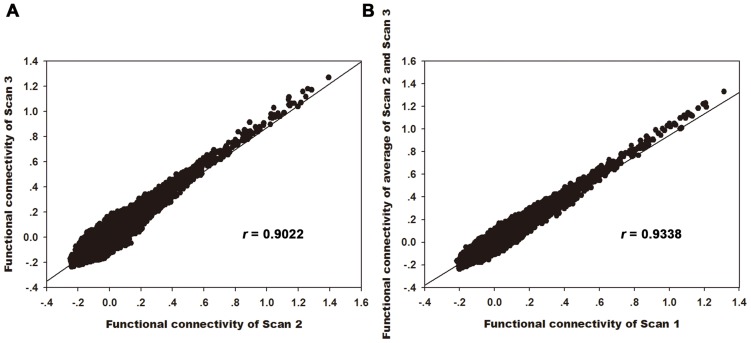
Consistency of functional connectivity strength of Broca's area in both short-term and long-term. Scatter plots of whole brain RSFC strength for Broca's area between Scan 2 and Scan3 (short-term) (A), and between Scan 1 and average of Scans 2 and 3 (long-term) (B), each dot in the graph represents a 3×3×3 mm^3^ voxel. Both of these two correlation coefficients are larger than 0.9, it revealed great consistency of FC strength in short-term and long-term.

Voxel-wise RSFC analysis demonstrated the whole brain correlation patterns related to the two seed ROIs which represented Broca's and Wernicke's areas. The patterns of the connectivity (Fig. S1) with the two seed ROIs were similar to those of the previous study [8]. The mean Fisher's *Z-*transformed RSFC maps across subjects were calculated for each scan. Fig. 2 shows the high spatial similarity across the whole brain between different functional scans (Scan 2 vs. Scan3: *r* = 0.9022; Scan 1 vs. average of Scan 2 and Scan 3: *r* = 0.9388).

### Test-retest reliability of the resting-state language network

**Figure 3:**
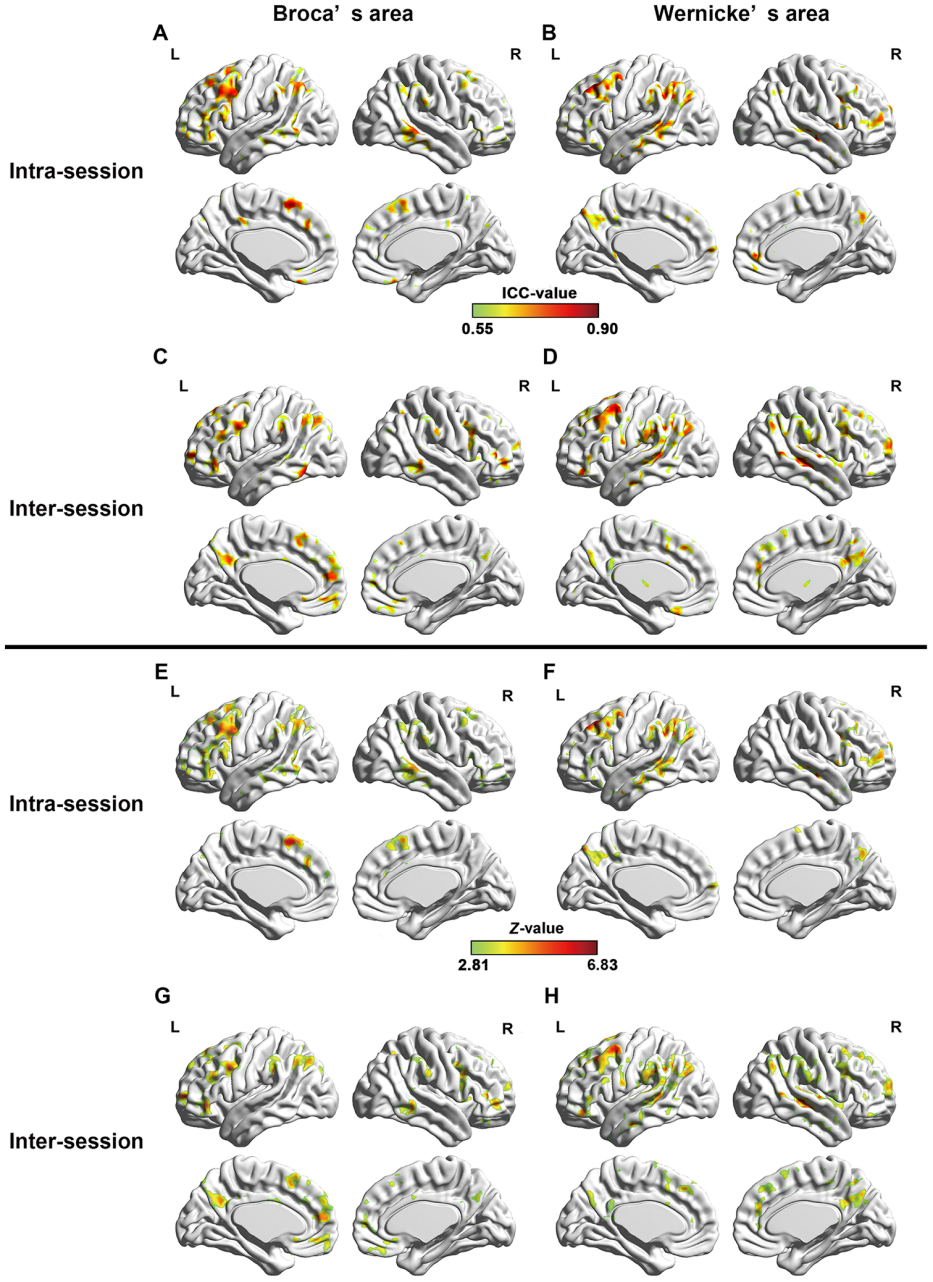
Test-retest reliability of language network. Short-term reliability maps (*Z* maps and ICC maps) for resting-state language network seeding at Broca's (A, E) and Wernicke's areas (B, F), and long-term reliability maps (*Z* maps and ICC maps) for resting-state language network seeding at Broca's (C, G) and Wernicke's areas (D, H). Only regions with high reliability are depicted (voxel level *p*<0.005, voxel size > = 200, corresponding to corrected *p_FWE_* <0.05). L and R represent left hemisphere and right hemisphere, respectively.

**Figure 4:**
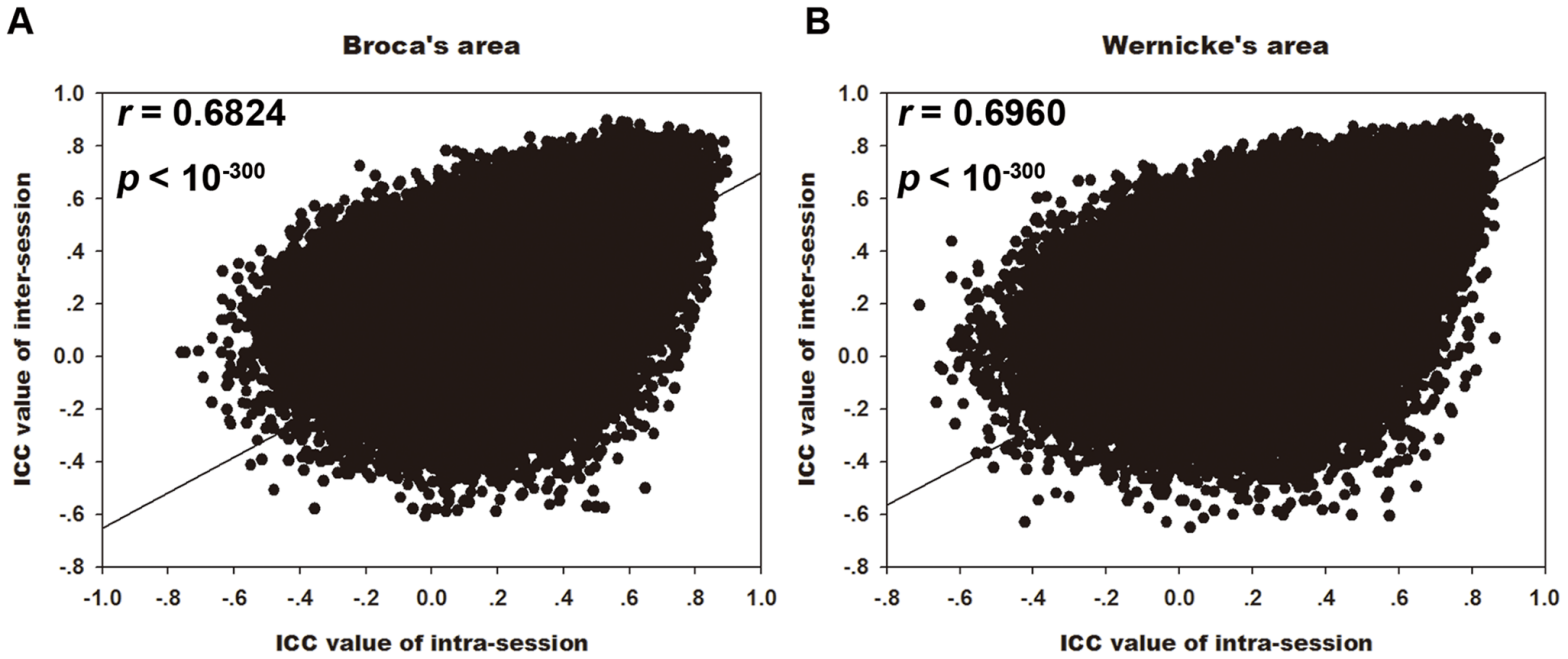
Relationship between short-term and long-term reliability of language network. Scatter plots between short- and long-term reliability among whole brain resting-state language network seeding at Broca's area (A) and Wernicke's area (B). Each dot in the graph represents a 3×3×3 mm^3^ voxel. Both of these two correlation coefficient are larger than 0.65, indicating short-term and long-term reliability are significantly correlated.

To evaluate the short- and long-term reliability of the RSFC, we calculated the intra- and inter-session ICC maps for each of the two ROIs (lBro, lWer) (Fig. 3, Table 1). The ICC maps of intra- and inter-session were spatially highly similar across the whole brain with each other for both seed ROIs (Fig. 4, Broca's area: *r* = 0.6824, *p*<10^−300^; Wernicke's area: *r* = 0.6960, *p*<10^−300^). For Broca's area, regions with high reliability were located at the bilateral frontal cortices, middle temporal gyrus (MTG) and inferior (IPL), left middle occipital gyrus (MOG), precuneus, and posterior cingulate gyrus. And for Wernicke's area, regions with high reliability located at the left supramarginal gyrus (SMG) bilateral MTG/inferior temporal gyrus (ITG), bilateral frontal cortices and bilateral IPL.

**Table 1:** Regions that showed high Intra- and inter-session reliability of functional connectivity with Broca's and Wernicke's area.

No.	Anatomical region	BA	MNI coordinates			Peak value		Cluster size (Voxels)
			x	y	z	*Z* value	ICC value	
**Intra-session reliability**								
*Broca's area*								
1	Left SFG/MFG and extend to cinglulate gyrus	8	−3	18	57	6.62	0.895	2664
2	Left IPL	39/40	−60	−42	42	5.26	0.817	953
3	Right IPL	40	48	−36	48	5.26	0.817	532
4	Right IFG	11	51	33	−12	5.00	0.796	437
5	Light MTG	21	−51	−48	−9	5.52	0.835	448
6	Right MTG	21	66	−39	0	5.37	0.825	344
*Wernicke's area*								
Z1	Left SMG, MTG, MFG and extend to precuneus	7/10	−48	−51	36	6.13	0.871	4815
2	Right MTG	21/22	57	−15	−12	5.45	0.830	263
**Inter-session reliability**								
*Broca's area*								
1	Bilateral SFG/MFG and also including left IFG and right cingulate gyrus	9	48	42	6	5.96	0.862	3820
2	Left IPL and extend to MOG	40	−36	−66	45	6.11	0.870	1025
3	Left Precuneus	7	−6	−54	30	4.97	0.795	457
4	Right IPL	40	33	−57	48	5.26	0.817	329
5	Right MTG	21	63	−45	−9	5.05	0.801	203
*Wernicke's area*								
1	Right STG, MFG/SFG and extend to IPL and precuneus	7	51	−30	3	6.15	0.872	3445
2	Left IFG/MFG and extend to precentral gyrus and right SFG	6	−42	6	57	6.76	0.901	2515
3	Left SMG, IPL and extend to ITG/STG	42	−48	−48	33	6.20	0.875	1937

Abbreviations: IFG  =  inferior frontal gyrus, MFG  =  middle frontal gyrus, SFG  =  superior frontal gyrus, IPL  =  inferior parietal lobule; ITG  =  inferior temporal gyrus, MTG  =  middle temporal gyrus, STG  =  superior temporal gyrus, SMG  =  supramarginal gyrus, MOG  =  middle occipital gyrus.

### Hemispheric asymmetry analysis of the resting-state language network

**Figure 5:**
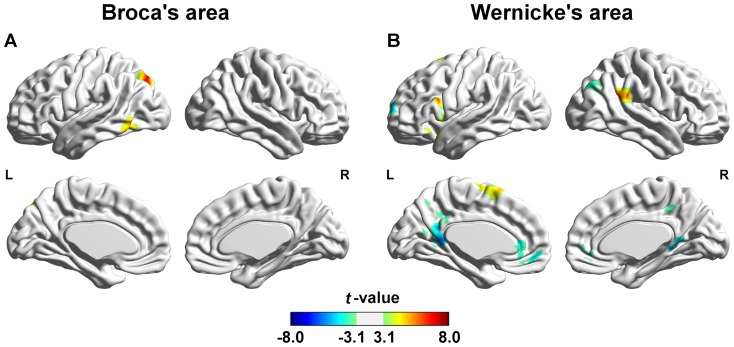
Functional lateralization of language network. Hemispheric asymmetry of functional connectivity patterns of Broca's area (A) and Wernicke's area (B). L and R represent left hemisphere and right hemisphere, respectively. The foci in the left hemisphere show significant asymmetric functional connectivity with their ipsilateral seeds, and the foci in the right hemisphere show significant asymmetric functional connectivity with their contralateral seeds. Results were statistically corrected (voxel level *p*<0.005, voxel size > = 200, corresponding to corrected *p_FWE_* <0.05).

The functional hemispheric asymmetry maps of Broca's and Wernicke's areas were shown in Fig. 5. Ipsilateral hemispheric asymmetry and contralateral hemispheric asymmetry were revealed in left hemisphere and right hemisphere respectively (Fig. 5). Significant functional asymmetry of Broca's area with ipsilateral hemisphere were shown in ITG/MTG and inferior occipital gyrus (IOG) (see Table 2). On the other hand, no cortical regions showed significant contralateral hemispheric asymmetry with Broca's area (see Fig. 5 and Table 2). Several regions showed significant ipsilateral asymmetry with Wernicke's area, which included precuneus, calcarine gyrus, IFG/MFG/superior frontal gyrus (SFG), ACC, insula cortex and SMA (see Table 2). Moreover, the Wernicke's area showed significant asymmetric FC with its contralateral hemisphere in several brain regions, such as precuneus, calcarine gyrus, SMG, STG, AG, MOG and SMA (see Table 2).

**Table 2:** Cortical regions revealed significant functional lateralization with Broca's and Wernicke's areas.

No	Anatomical regions	BA	MNI coordinates			Peak *Z* value	Cluster size (Voxels)
			x	y	z		
**Broca's area**							
1	Left ITG/MTG and extend to IOG	37/20	−48	−63	−3	7.25	359
2	Left IPG/SPG and extend to MOG	7/9	−25	−77	45	7.90	276
**Wernicke's area**							
1	Bilateral precuneus and extend to calcarine gyrus	31/7	9	−42	0	−7.13	662
2	Left MFG/SFG and extend to bilateral ACC	10/32	−27	63	12	−6.57	477
3	Left IFG and insula	47/38	−48	15	21	6.37	415
4	Right SMG and STG	40	57	−45	24	7.60	332
5	Right AG and MOG	39/19	27	−54	30	−5.43	313
6	Bilateral SMA	6	0	−3	69	6.07	264

Abbreviations: ITG  =  inferior temporal gyrus, MTG  =  middle temporal gyrus, STG  =  superior temporal gyrus, IOG  =  inferior occipital gyrus, MOG  =  middle occipital gyrus, IPG  =  inferior parietal gyrus, SPG  =  superior parietal gyrus, IFG  =  inferior gyrus, MFG  =  middle frontal gyrus. SFG  =  superior frontal gyrus, ACC  =  anterior cingulate cortex, AG  =  angular gyrus, SMG  =  supramarginal gyrus, SMA  =  supplemental motor area.

**Figure 6:**
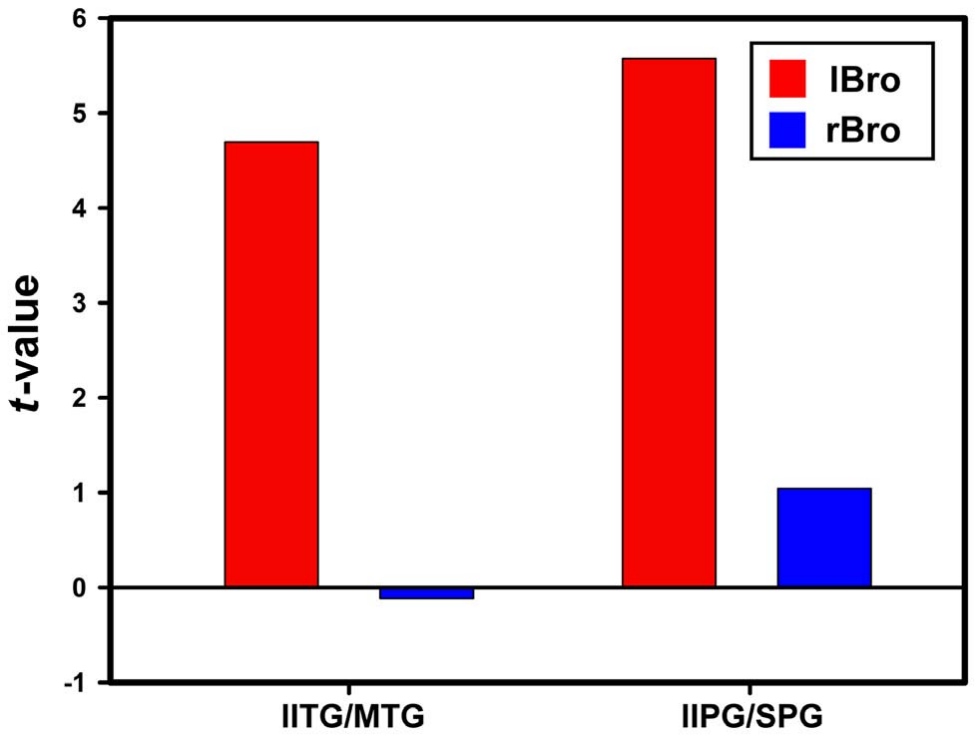
Average connectivity strength in the regions with significant functional hemispheric asymmetry of Broca's area. Red color denotes connectivity strength computed using left Broca's area as seed; blue color denotes connectivity strength computed using right Broca's area as seed. Prefix: l-left, r-right, b-bilateral. Abbreviation: ITG-inferior temporal gyrus, MTG-meddle temporal gyrus, IPG-inferior parietal gyrus, SPG-superior parietal gyrus.

**Figure 7:**
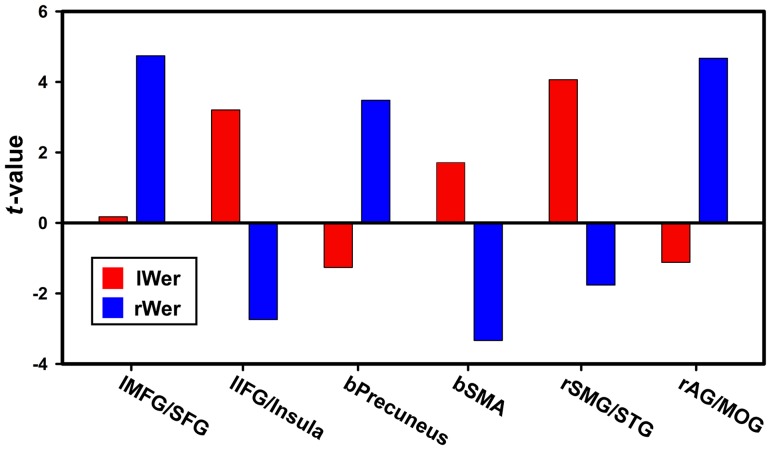
Average connectivity strength in the regions with significant functional hemispheric asymmetry of Wernicke's area. Red color denotes connectivity strength computed using left Wernicke's area as seed; blue color denotes connectivity strength computed using right Wernicke's area as seed. Prefix: l-left, r-right, b-bilateral. Abbreviation: MFG-middle frontal gyrus, SFG-superior frontal gyrus, SMA-supplemental motor area, SMG-supramarginal gyrus, STG-superior temporal gyrus, AG-angluar gyrus, MOG-middle occipital gyrus.

To further evaluate the RSFC strength within each region that revealed significant hemispheric asymmetry, mean *t* values within each significant cluster were extracted from one-sample *t* map of left seed and LR-flipped one-sample *t* map of the right seed. Fig. 6 shows the functional asymmetry patterns of Broca's area and Fig. 7 demonstrates those of Wernicke's area. It was shown that both of those two regions had greater RSFC strength with lBro than with rBro. The resting-state functional connectivity of Broca's area was leftward lateralized. On the contrary, RSFC of Wernicke's area was almost rightward lateralized. Four (ipsilateral MFG/SFG, bilateral precuneus and SMA, contralateral AG/MOG) out of six clusters showed greater connectivity strength with rWer than lWer.

### Temporal reliability of hemispheric asymmetry of resting-state language network

**Figure 8:**
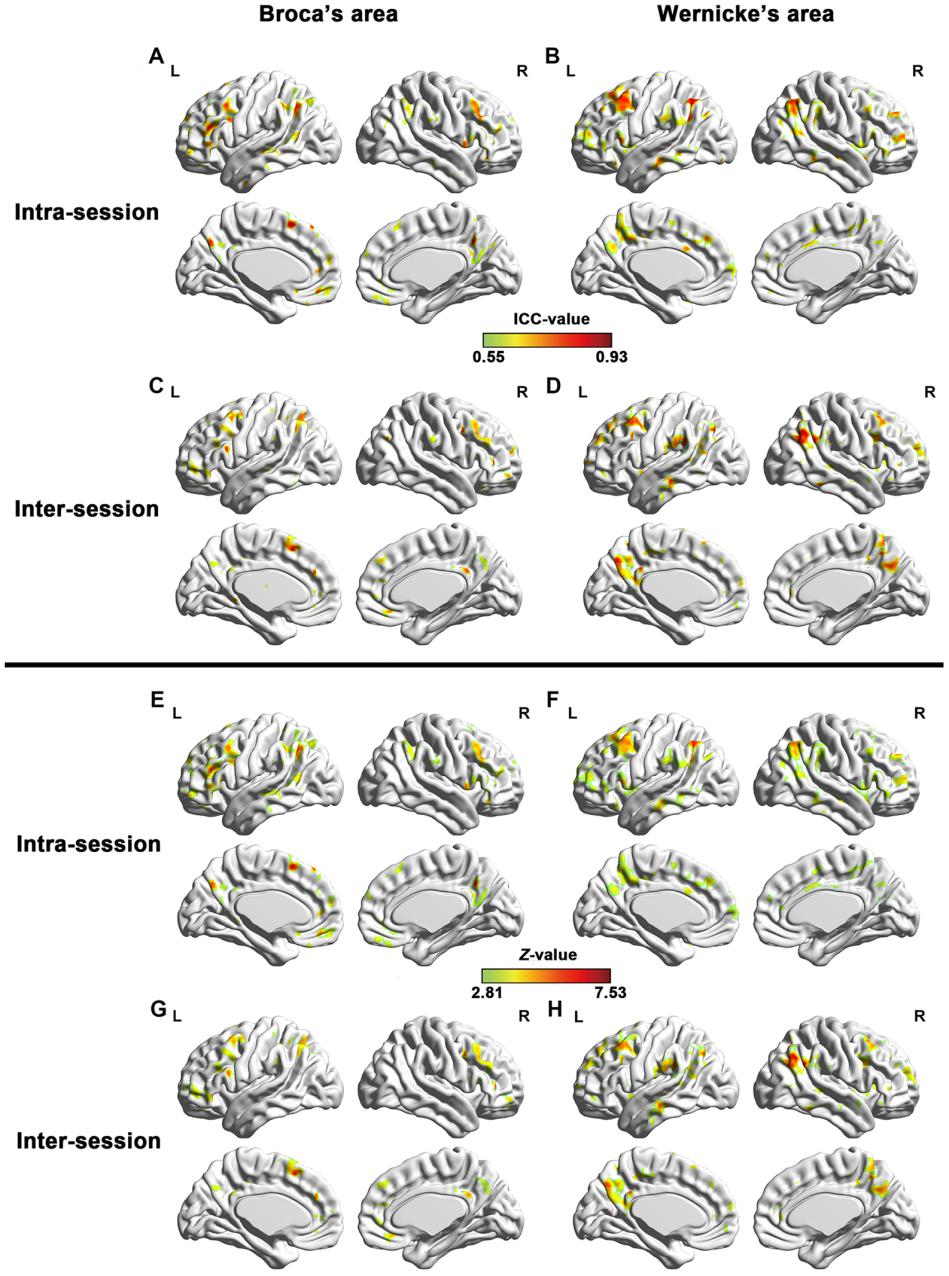
Test-retest reliability of functional lateralization of language network. TRT reliability of functional asymmetry (*Z* maps and ICC maps) of both Broca's (A, C, E, G) and Wernicke's (B, D, F, H) areas. Both intra-session (A, B, E, F) reliability and inter-session (C, D, G, H) reliability areas shown. Only regions with high reliability are depicted (voxel level *p*<0.005, voxel size > = 200, corrected *p_FWE_* <0.05). L and R represent left and right hemispheres, respectively.

To assess TRT reliability of functional hemispheric asymmetry of resting-state language network seeding at both Broca's and Wernicke's areas, we computed both inter- and intra-session voxel-wise ICC of AI of those two regions. Intra- and inter-session ICC maps and the converted *Z*-maps were shown in Fig. 8. Regions with significant temporal stability were listed in Table 3. We can see that functional asymmetry maps of both seed ROIs revealed high intra- and inter-session reliability in language related brain regions. Areas with significant inter-session ICC for AI of Broca's area located at bilateral IFG/MFG/ACC/MCC and precuneus, and left SFG/IPG/SPG and SMA. Regions with significant reliability for AI of Wernicke's area mainly located at frontal cortex (bilateral IFG/MFG/SFG) and temporal cortex (bilateral ITG/MTG/STG). In addition, bilateral insula cortex and ACC/precuneus showed great reliability of functional lateralization with Wernicke's area. Intra-session ICC maps were similar with inter-session ones.

**Table 3:** Cortical regions revealed significant test-retest reliability of functional lateralization

No	Anatomical regions	BA	MNI coordinates			Peak value		
			x	y	z	*Z* value	ICC value	Cluster size (Voxels)
**Intra-session reliability**								
*Broca's area*								
1	Bilateral IFG/MFG/SFG and extend to SMA and ACC	10/8/9	−3	15	57	6.02	0.865	2721
2	Left MTG/STG and IPG/SPG	40/7/21	−36	−63	−39	6.15	0.872	1188
3	Bilateral precuneus and extend to MCC and calcarine gyrus	7/31	6	−54	39	6.37	0.883	480
4	Bilateral MFG/SFG and extend to ACC	11	−3	45	−15	5.46	0.831	460
5	Right SMG, IPG and extend to postcentral gyrus	40	27	−39	51	5.31	0.821	375
*Wernicke's area*								
1	Left IFG/MFG/SFG and IPG, also include STG/MTG and bilateral MCC and precuneus	7/40/10	−45	−51	36	7.50	0.927	4960
2	Right IFG/MFG/SFG	10/9/47	42	12	−6	5.95	0.861	1147
3	Right MTG, SMG, AG and extend to IPG	40/39	51	−60	48	5.88	0.857	1127
**Inter-session reliability**								
*Broca's area*								
1	Left IFG/MFG/SFG and extend to SMA	6	−6	18	51	5.91	0.859	1203
2	Left SPG/IPG and AG	40	−39	−57	48	6.28	0.879	672
3	Bilateral MFG and ACC	10/9/32	−3	42	27	5.39	0.826	523
4	Right IFG/MFG	9/8	51	21	36	5.36	0.824	492
5	Bilateral MCC and precuneus	31/7	15	−45	30	5.06	0.802	360
*Wernicke's area*								
1	Left ITG/MTG/STG SMG and bilateral precuneus	7/40/31	−3	−45	36	5.77	0.851	2415
2	Left MFG/SFG precentral gyrus and bilateral ACC	9/10/8	−51	15	33	5.81	0.853	1566
3	Right AG, SMG and extend to STG/MTG	40/39	60	−51	30	5.83	0.854	850
4	Right SFG/MFG/IFG	10/8/9	42	15	54	5.33	0.822	662
5	Right IFG and insula	13	51	18	−6	6.11	0.870	452
6	Left IFG insula and STG	13	−51	21	−9	5.55	0.837	311
7	Right ITG/MTG/STG	21	63	−45	−18	5.58	0.839	275

Abbreviations: IFG  =  inferior frontal gyrus, MFG  =  middle frontal gyrus, SFG  =  superior frontal gyrus, ITG  =  inferior temporal gyrus, MTG  =  middle temporal gyrus, STG  =  superior temporal gyrus, IPG  =  inferior parietal gyrus, SPG  =  superior parietal gyrus, ACC  =  anterior cingulate gyrus, MCC  =  middle cingulate gyrus, AG  =  angular gyrus, SMG  =  supramarginal gyrus, SMA  =  supplemental motor gyrus.

### The impact of global signal regression on TRT reliability of the resting-state language network

**Figure 9:**
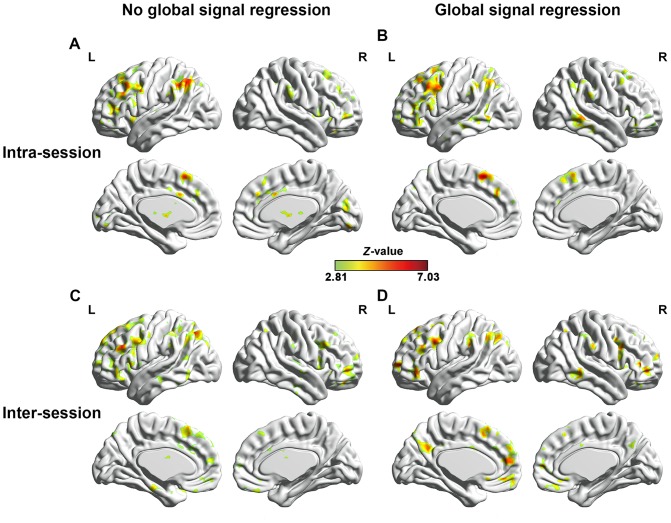
The impact of GSR on TRT reliability of language network. Comparison of ICC maps of RSFC of Broca's area between with (C, D) and without (A, B) global signal regression. *Z*-transformed intra-class correlation coefficient (ICC) is used to assess TRT reliability. Only regions with high reliability are depicted (voxel level *p*<0.005, voxel size > = 200, corresponding to corrected corresponding to corrected *p_FWE_* <0.05. L and R represent left and right hemispheres, respectively.

**Figure 10:**
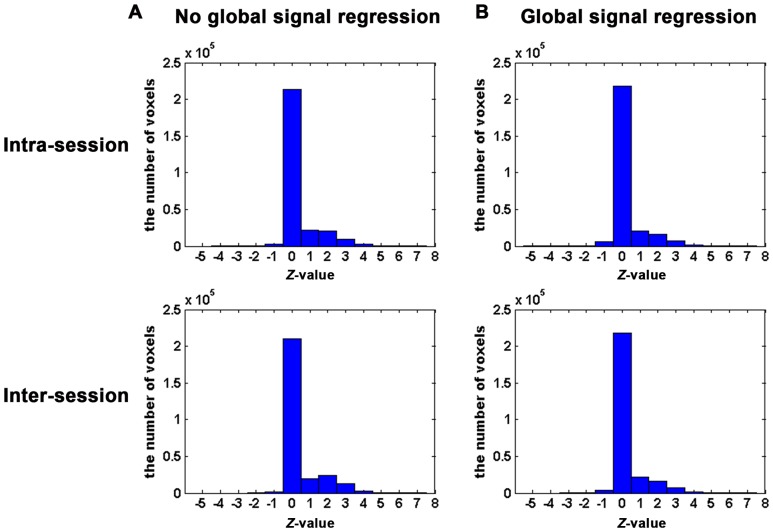
The histograms of ICC values across the whole brain voxels with (B) and without (A) GSR.

**Figure 11:**
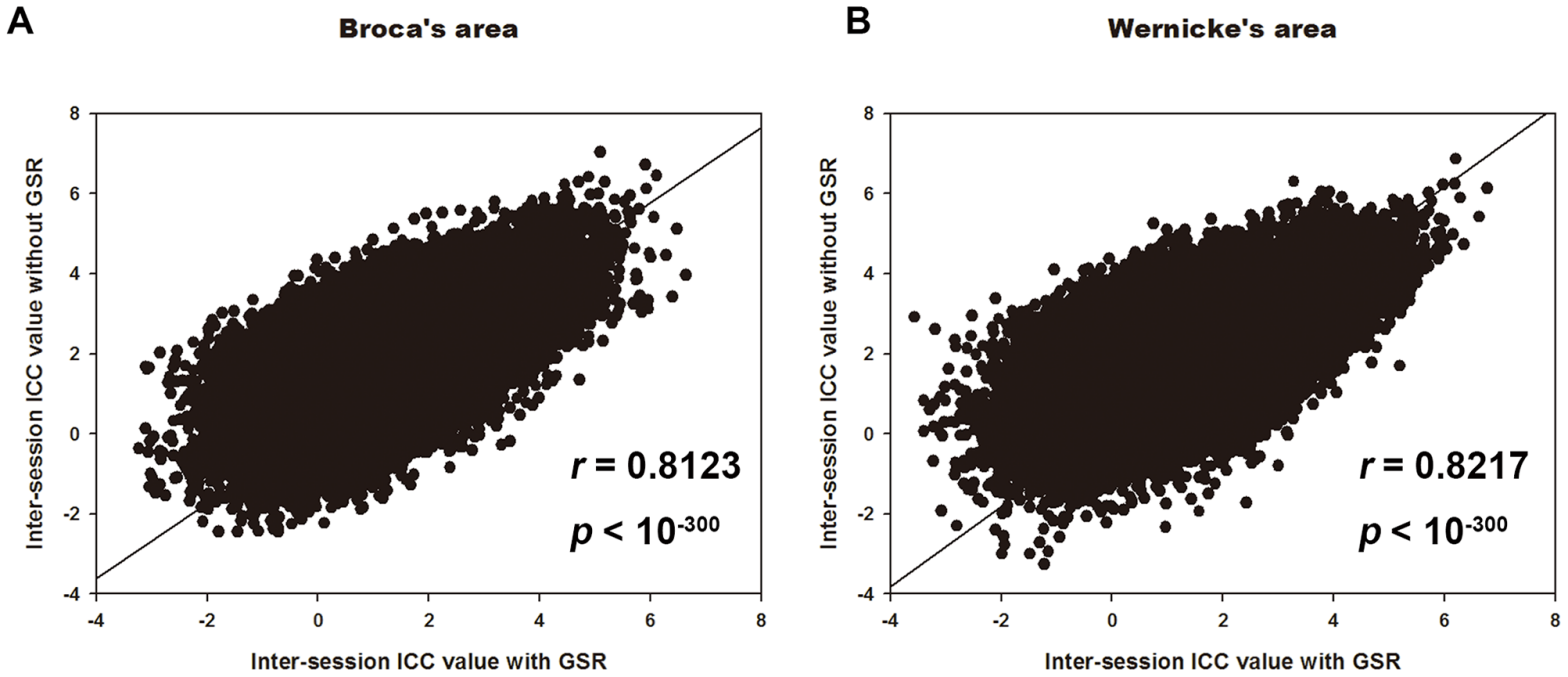
Relationship between ICC values of two regression conditions. Scatter plots of whole brain RSFC inter-session ICC values between with and without global signal regression. Both Broca's (A) and Wernicke's areas (B) are depicted. Each dot in the graph represents a 3×3×3 mm^3^ voxel. Both of these two correlation coefficient are larger than 0.8. It reveals high correlation of temporal reliability no matter the global signal was regressed or not.

To evaluate the impact of GSR on TRT reliability of the resting-state language network, the ICC patterns identified with and without GSR for Broca's and Wernicke's areas were computed respectively. Fig. 9 shows the ICC maps of Broca's area with and without GSR. Both the intra- and inter-session ICC maps with GSR demonstrated similar patterns to those without GSR. Similar findings were observed on ICC maps of Wernicke's area (data not shown). In addition, the histograms of ICC values with and without GSR shows that the patterns of ICC value distribution are similar to each other (Fig. 10). Furthermore, Fig. 11 shows the scatter plots of the whole brain inter-session ICC values of RSFC between with and without GSR. The voxel-wise ICC computed with GSR were highly consistent with values which were computed without GSR. We further compared between ICC values with and without global signal regression for each voxel [51]. However, no significant differences were shown for either intra- or inter-session reliability with FWE correction (*p_FWE_* <0.05) or with much looser threshold (p<0.01, voxel size > = 40). In addition, Fig. 11 shows the scatter plots of the whole brain inter-session ICC values of RSFC between with and without GSR. The voxel-wise ICC values computed with GSR were highly consistent with values which were computed without GSR.

## Discussion

We calculated the whole brain RSFC and the functional asymmetry for Broca's and Wernicke's areas respectively and evaluated their short- and long-term reliability. In addition, the impact of global signal regression on test-retest reliability of resting-state language network was investigated. We found that there was a highly temporally reliable, both short- and long-term, language network during resting state. Moreover, we found the RSFC of Broca's area was mainly left lateralized, while that of the Wernicke's area was right lateralized. In addition, both Broca's and Wernicke's areas revealed high inter- and intra-session reliability for functional asymmetry in most brain regions. Finally, we showed that TRT reliability of the resting-state language network was little impacted by the GSR.

### Functional connectivity of resting-state language network and its test-retest reliability

The RSFC patterns based on Broca's and Wernicke's areas were highly similar across three scans. This resting state language network was similar to that of language comprehension and production network which was reported in the reviews [1], [52], [53] and additionally consistent with a recent study that showing the RSFC of language processing based on 970 subjects [8]. The language processing networks were comprised of two streams. A ventral stream, including structures in the superior and middle portions of the temporal lobe, is involved in processing speech signals for comprehension and a dorsal stream, including regions in the posterior frontal lobe and the posterior dorsal-most aspect of the temporal lobe and parietal operculum, is involved in translating acoustic speech signal into articulatory representations in the frontal lobe [52], [54].

Those negative correlation pattern might demonstrate the functionally temporal segregation of auditory and visual perception and that of speech comprehension and production [8]. In a recent study, two distinct networks during the comprehension of story endings were revealed: positive correlations in areas usually involved in language processing and memory for language, and negative correlations in sensory, motor and visual areas, indicating that weaker activity in the latter regions is conducive to better memory for linguistic content [55].

The resting-state language networks were temporally (both short- and long-term) reliable. The highly reliable regions were mainly located at bilateral frontal cortices, IPL, MTG and precuneus. The highly reliable regions were those typically observed to be coactive during task-based studies [54], [56]–[58] and similar to the resting-state language network mentioned above. In addition, the ICC maps of Broca's and Wernicke's areas were similar to each other.

imilar to other TRT studies of RSFC [11], [39], [41], [43], [59]–[65], the RSFC of language network shows excellent temporal reliability. Temporal TRT reliability and stability across institutions [8] of the resting-state language network give us a corroborative evidence to establish the researches on language network using the resting-state data. With excellent both short- and long-term TRT reliability, RSFC of language network could be potentially applied in longitudinal monitoring, such as disease progression and/or treatment effect across time in patient populations [62] with deficits in language system [61]. In previous studies, RSFC were used to explore the pathology of mental diseases [66]–[71], and resting-state language network has already used to explore the differences between normal subjects and patients with language disorders [14], [18], [72]. These researches were based on the hypothesis that the resting-state language network is temporal reliable and stable. Tomasi and his colleagues demonstrated that this network was stable across institutions [8] and our results showed the temporal reliability of the resting-state language network. These provided methodology basis for future cross-culture and clinical studies of language network.

### Lateralization of language network and its Test-retest reliability

We examined the functional lateralization of resting-state language network using Broca's and Wernicke's areas as seeds. The functional network of Broca's area was almost leftward lateralized (see Fig. 8). This finding was in correspondence with previous resting-state fMRI studies and supported the left hemisphere dominance of the language network [8], [73]. Significant leftward lateralization of RSFC with Broca's area also located at IPL. Hickok and his colleagues proposed the “dual stream model”, which indicated dorsal and ventral streams between Broca's and Wernicke's areas [52], [54]. Recently, Catani and his colleagues asserted the existence of two dorsal pathways, a direct one and an indirect one [61]. The indirect one connected Broca's and Wernicke's areas through fibers passing through IPL [61]. Functional asymmetry of connectivity between Broca's area and IPL in the present study might suggest the presence of this indirect pathway. Moreover, the anatomical leftward lateralization of this indirect dorsal pathway was also proved [30].

However, rightward asymmetry was found in most regions within functional network of Wernicke's area. It was not beyond our expectation and evidences of this rightward lateralization can be found in previous studies. Rightward lateralization long-range connectivity density of the anterior Wernicke's region was revealed by Tomasi and Volkow [8], [74]. Structural proof of this rightward lateralization also existed. Compared with left hemisphere, the right hemisphere has larger extend of Silvian fissure, which is adjacent to Wernicke's area [75], [76]. What's more, increasing evidence suggests the right hemisphere seems to process figurative language [77]. However, evidences asserted that high level of acoustic noises generated by EPI acquisition may also attribute to this rightward lateralization because of rightward lateralized auditory motion perception [78], [79]. As a result, further work should be done in this point.

TRT reliability of functional hemispheric asymmetry of language networks was also assessed via ICC. We showed that widely spread cortical regions revealed significant both intra- and inter-session temporal reliability of functional asymmetry. The significant reliable regions included frontal cortices, temporal cortices and precuneus. These cortical areas were all within language network and default mode network. It demonstrated that functional asymmetries of language networks were both short- and long-term temporally stable. Considering that the lateralization of language related networks might be a biomarker of mental disorders, such as schizophrenia, our findings suggest its potentially clinical application in tracing longitudinal effect of treatment and training.

### Impact of GSR on TRT reliability of language network and its lateralization

The ICC patterns with and without GSR were similar to each other, though GSR would impact the RSFC patterns at both the individual and group level. In previous TRT studies, some preprocessed the resting-state data with [11], [39], [40] and some others without performing GSR [63], and both preprocessing strategies yielded high TRT of resting-state networks. Here we showed that the temporal reliability of major language network in resting state was not impacted by GSR. This additionally added evidence that the language network during resting state was reliable and stable.

### Limitations and future works

The current study is not without limitations. Firstly, a recent study showed that reliability of RSFC can be greatly improved by increasing the scan lengths from 5 min up to 13 min [59]. In our study, the scan length was shorter than 13 min, though the reliability of the resting-state language network was high for both intra- and inter-session measurements. Secondly, physiological signals were not given in the dataset. Physiological noise is known to be proportional to MRI signal strength [80]. Subtle changes in either breathing pattern or cardiac pulse rate could alter BOLD fMRI signals. Resting-state fluctuations in BOLD fMRI signals could possibly not be related to underlying neuronal activations of interest but instead contaminated by physiological noises. However, impact of cardiac and respiratory-related processes on resting-state signals within gray matter appears to be relatively small [81]. Thirdly, the TRT for atypical populations would be more useful than that for the normal ones, as TRT reliability could help to explore disease progression and/or treatment effect across time in patients with brain disorders. TRT reliability and laterality of resting-state language network for atypical populations should be investigated in future studies. Lastly, for future works, other resting-state analysis approaches could be used to investigate the TRT reliability of resting-state language network, such as principal component analysis [82], singular value decomposition [83], independent component analysis [84]–[86] and clustering 87–90.

## Conclusion

In this study, we established seed-based analysis of language network and its functional asymmetry during resting state, and investigated TRT reliability of the language network. We found there was a highly reproducible language network during resting state. Furthermore, we showed that the RSFC of Broca's area was left lateralized, while that of the Wernicke's area was right lateralized. Moreover, both Broca's and Wernicke's areas revealed high inter- and intra-session reliability of functional asymmetry of RSFC in most brain regions. In addition, the reliability of the resting-state language network was little affected by whether performing GSR during preprocessing.

## Supporting Information

Figure S1 **Functional connectivity patterns of Broca's and Wernicke's areas.** The whole brain RSFC maps of Broca's (A) and Wernicke's areas (B). Results were statistically corrected (voxel level *p*<0.005, voxel size > = 200, corresponding to corrected *p_FWE_* <0.05). L and R represent left hemisphere and right hemisphere, respectively.
(TIF)

Text S1 **Derivation of equation for testing significance of ICC.**
(DOCX)
