# Supplementary figures and images for: Temporal Reliability and Lateralization of the Resting-State Language Network
Source: PLoS One. 2014 Jan 24;9(1):e85880. doi: 10.1371/journal.pone.0085880 (PMC3901661; doi:10.1371/journal.pone.0085880)

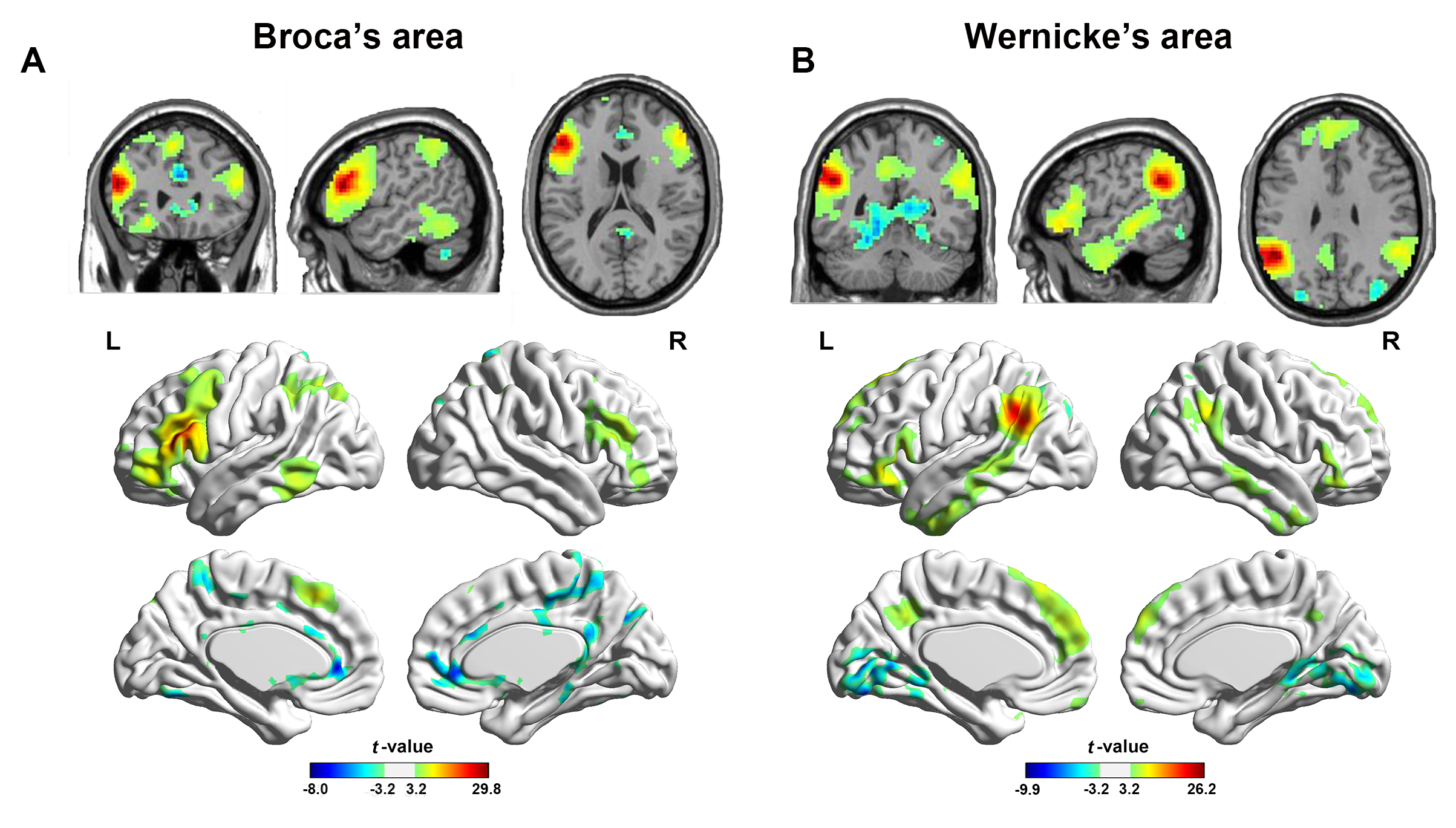

Supplement: Figure S1 — Functional connectivity patterns of Broca's and Wernicke's areas. The whole brain RSFC maps of Broca's (A) and Wernicke's areas (B). Results were statistically corrected (voxel level p<0.005, voxel size > = 200, corresponding to corrected pFWE <0.05). L and R represent left hemisphere and right hemisphere, respectively. (TIF) [file pone.0085880.s001.tif]
