# Supplementary material for: Temporal Reliability and Lateralization of the Resting-State Language Network
Source: PLoS One. 2014 Jan 24;9(1):e85880. doi: 10.1371/journal.pone.0085880 (PMC3901661; doi:10.1371/journal.pone.0085880)
Supplement: Text S1 — Derivation of equation for testing significance of ICC. (DOCX) [file pone.0085880.s002.docx]

We derived equation (3) as follows. For interclass correlation, such as Pearson’s correlation (*r*), we transform *r* to *z* using Fisher’s transformation:

$$z=\frac{1}{2}\ln\frac{1+r}{1-r}$$

However, for intraclass correlation, we use the formula [[1](#_ENREF_1)]

$$z=\frac{1}{2}\ln\frac{1+(k-1)r}{1-r}$$

Where k is the number of observation made on each subject of measurement, and k =2 in the current study. The mean of the above statistic is zero and the variance [[1](#_ENREF_1)] is

$$\sigma^{2}=\frac{k}{2(n-2)(k-1)}$$

Where n is the number of subjects of measurement, and k is the number of observation made on each subject of measurement.

To transform *z* in to *Z* scores with standard normalization distribution:

$$Z=\frac{z-0}{\sqrt{\frac{k}{2(n-2)(k-1)}}}$$

Replace *z* with $\frac{1}{2}\ln\frac{1+(k-1)r}{1-r}$and k with 2,

$$Z=\frac{\frac{1}{2}\ln\frac{1+r}{1-r}}{\sqrt{\frac{2}{2(n-2)(2-1)}}}$$

*Z* scores could be transformed into the following,

$$Z=\frac{\sqrt{n-2}}{2}\ln\frac{1+r}{1-r}$$

In the end, we got the final equation of *Z* scores of ICC values,

$$Z=\sqrt{n-2}\tanh^{-1}(r)$$

Reference:

1. McGraw KO, Wong S (1996) Forming inferences about some intraclass correlation coefficients. Psychological methods 1: 30.
